# Embodied Learning in Physical Activity: Developing Skills and Attunement to Interaction

**DOI:** 10.3389/fspor.2022.795733

**Published:** 2022-01-31

**Authors:** Susanne Ravn

**Affiliations:** Research Unit Movement, Culture and Society, Department of Sport Science and Clinical Biomechanics, University of Southern Denmark, Odense, Denmark

**Keywords:** embodiment, physical activity, practice, learning, skills, interaction, attunement

## Abstract

This article focuses on embodied learning and how it develops through the practice of a physical activity. It aims to clarify fundamental theoretical aspects of the development that takes place when practitioners enhance their way of participating in the activity. Pursuing this aim, I draw on the phenomenological description of embodiment processes and argue, that despite differences in the inherent logic and motivation for engaging in different kinds of physical activities, a phenomenologically based understanding of skills constructively helps describe the development acquired through practicing a physical activity. I thereby argue that descriptions of how skills are incorporated and exercised are relevant to analyses of embodied learning processes taking place in sporting as well as non-sporting activities, including recreational mountain-biking, expressive and creative dance activities, and improvisational practices. In continuation of this argument, I also suggest that practitioners' capability to attune to the interactions of the activity contribute yet another theoretical aspect we should consider, when aiming at describing the embodied learning that takes place in different kinds of physical activities. This suggestion finds support in recent work on action and interaction.

## Introduction

In a recent review on pedagogies of embodiment in physical education, the notion of embodiment is presented as a concept that broadens the focus on the body and facilitates an understanding of the bodily engagement beyond what is referred to as “the dualistic natural scientific point of view” (Aartun et al., 2020). Based on a review of 42 articles, Iselin Aartun and co-authors highlight that pedagogies which specifically address embodiment perspectives either aim to enable critical reflection or explore novel movements. The latter pedagogical aim is closely connected to the intention of creating pedagogical spaces for students in which to explore their “embodied identity,” “listen to the bodies they are” and potentially to be empowered in relation to their self-understanding when it comes to evaluating their embodied capabilities (Aartun et al., 2020). In accordance with the way the notion embodiment is used in the articles reviewed, recent discussions of physical education tend to use the notion of embodiment to highlight the potential of activities aimed at offering learning opportunities based on explorations and reflections (e.g., Larsson and Karlefors, 2015; Standal and Aggerholm, 2016; Aggerholm et al., 2018). Consequently, pedagogically related discussions of embodied learning in physical education are rarely connected to the acquisition and performance of sporting skills. As I will indicate in this article, the way embodied learning is put to use in relation to the actual practice of physical activities thereby hardly reflects the way processes of embodiment and incorporation of skills are discussed in classical phenomenology (e.g., Merleau-Ponty, 1962, 1963) and contemporary phenomenologically grounded work (e.g., Leder, 1990; Gallagher, 2020). Philosopher of sport Breivik's works (Breivik, 2008, 2014, 2016a,b) present an exception to this tendency. In his work, Breivik explicitly suggests how different phenomenological grounded descriptions of skills can be understood in relation to actual physical activities (Breivik, 2008, 2016a,b). However, in so doing, Breivik focuses only on sporting activities. Until now, his description of skills has not been discussed or related to activities beyond the performative environment of sportified competitions.

This article focuses on embodied learning and how it develops through the practice of a physical activity. It aims to clarify fundamental, theoretical aspects of the development that takes place when practitioners enhance their way of participating in an activity. Pursuing this aim, I draw on the phenomenological description of embodiment processes and argue that despite differences in the inherent logic and motivation (Engström et al., 2017) for engaging in different kinds of physical activities, a phenomenologically based understanding of skills constructively contributes to describing the development acquired through the practice of physical activity. Thus, I argue that descriptions of how skills are incorporated and exercised are relevant to analyses of the embodied learning that takes place in sports as well as different kinds of non-sporting activities, including recreational mountain-biking, expressive and creative dance activities, and improvisational practices. In continuation of this argument, I also suggest that practitioners' capability to attune to the interactions of the activity contribute yet another theoretical aspect we should consider, when aiming at describing the embodied learning that takes place in different kinds of physical activities. Throughout the argumentation I draw on experience-based descriptions of practices.

I begin the following sections by giving a brief introduction to embodiment drawing on the phenomenological descriptions presented in Merleau-Ponty's (1962), Drew Leder (1990), and Breivik (2016a,b) work. This section presents a theoretical ground for using experience-based descriptions of “learning to mountain-bike” to exemplify and further expand on phenomenological descriptions of skills. Next, I discuss expressive and creative dance activities and how the phenomenological account of skills can be useful in better understanding the embodied learning at stake in these kinds of physical activities. Finally, I turn to improvisational practices to indicate that these kinds of practices specifically highlight yet another aspect of embodied learning: the capability to attune to interaction, an aspect of embodied learning which I suggest works in a complementary manner to the development of skills. In this latter part, I specifically draw on philosopher Shaun Gallagher (Gallagher, 2020) recent work on action and interaction.

Throughout the argumentation and examples of practices presented, I use the term ‘physical activity' in accordance with Piggin (2020), who argues that we should indeed involve a “holistic definition.” Thus, I use the notion of physical activity to address a wide range of activities in which “people [are] moving, acting and performing within culturally specific spaces and contexts, and influenced by a unique array of interests, emotions, ideas, instructions and relationships” (Piggin, 2020). Further, I use the notions “practitioners” and “practicing” to indicate that the people involved in the activity in focus for the discussion are expected to strive for something, such as becoming better at participating, mastering certain skills, being able to handle a state of mind and/or developing a specific kind of sensorial awareness while moving.

## A Phenomenological Approach to Embodied Learning And the Incorporation of Skills

Based on the work of Edmund Husserl, phenomenology contributes fundamental accounts for how the body is much more than a physical condition for our existence. It is the basis for our “being in the world” and fundamental to our experiences. Husserl's original descriptions of kinesthesia and pre-reflective self-awareness are central for his account of the “unthematic, pre-reflective lived body-awareness” that accompanies and conditions subjective experience (Zahavi, 2003, p. 101). This pre-reflective body-awareness is to be understood as the functioning body and grounds any of the thematization and objectification of the body we might engage in—whether this engagement is connected to scientific laboratory experiments or analyses of activities in everyday life activities. The description of the unthematic, pre-reflective lived body-awareness is also central to Husserl's later descriptions of subjectivity and the world as inseparable. As described in Zahavi's discussions of Husserl's work: “The body is not first given for us and subsequently used to investigate the world. On the contrary, the world is given to us as bodily investigated, and the body is revealed to us in this exploration of the world,” (Hua 5/128, 15/287, according to Zahavi, 2003, p. 105).

Based on Husserl's work, Merleau-Ponty describes how perception and movement are fundamental to the ongoing process of revealing to us what the body is and can be (Ravn, 2009, ch. 3). Throughout *Phenomenology and Perception*, Merleau-Ponty (1962, p. 110, p. 152, p. 270) emphasizes that movement and perception form part of a whole and accounts for perceptual fields, i.e., the ways in which perception-action possibilities unfold according to an experiential based horizon. Perceptual fields open through the body with its various biological predispositions and, not least, *as the body is lived*. As is often quoted with explicit reference to Merleau-Ponty, it is never our objective body that we move, but our phenomenal—or lived—body. We exist in the world in and through a body with a certain kind of interwoven register of perceptual fields. Perceptual fields are never static but in process. In that sense, my body is not just a lived but a living body, as recently emphasized by phenomenologist Heinämaa (2021). Merleau-Ponty outlines that the phenomenal body can be compared to a nexus of living meanings (Heinämaa, 2021, p. 150) and that it is also our general instrument of “comprehension” (Merleau-Ponty, 1962, p. 235), an “instrument”^1^ which functions in an interactive fashion and in a process of change, just as comprehension goes on pre-reflectively.

The notion of embodiment addresses this phenomenal body and, thus, the ongoing processes constituting and (re)shaping our being in the world. Anthropologist Csordas (1993, 2011) has coupled the phenomenological accounts of embodiment to analyses of how enculturation is conditioned on embodiment processes. With close reference to phenomenology, he refers to embodiment as the way in which “perceptual experiences and modes of presence and engagement in the world” have taken and take shape (Csordas, 1994, p. 12, quoted in Csordas, 2011, p. 137). Csordas' analyses focus on the aspects of embodiment processes underlying the way we see, sense and relate to things, stories and other people on cultural terms, and which take place in the background of our experiential life. However, processes of embodiment can also take place based on conscious and active decisions of the subject, as is the case when the subject engages in practicing a physical activity and strive for some kind or degree of development, for example. Drawing on Csordas' description of embodiment, the changes in “perception,” “presence,” and “engagement” are deliberately aimed for through practicing. It is primarily this latter level of embodiment processes that I address using the notion “embodied learning.”

Leder (1990) has among other things addressed how skill acquisition is considered an embodied process of “incorporation.” Following Leder's descriptions:

A skill is finally and fully learned when something that once was extrinsic, grasped only through explicit rules or examples, now comes to pervade my own corporeality. My arms know how to swim, my mouths can at last speak the language. […]–when I finally master a foreign language, it is as if all the instruction, study, and practice finally have sunk right into my tongue. A skill has been incorporated into my bodily “I can” (1990, p. 31) and part of the register of one's perceptual fields (Leder, 1990, pp. 30–31).

The incorporation of skills potentially changes our way of seeing things and acting in the world. As Leder exemplifies after having incorporated swimming skills, the pool simply looks different. It now presents a possible field for certain actions, interactions and experiences.

In his philosophical work on skills in sporting activities, Breivik (2016a) echoes Leder's account of skill being based in processes of incorporation. Breivik also emphasizes that we should distinguish between ability and skill, however. Whereas, ability defines a natural capacity that we have without having engaged in special or purposeful training, a skill is acquired through a degree of deliberate engagement when practicing an activity. Breivik's point here is that skills come with practice and training *and* admits degrees^2^. Presenting such a distinction, Breivik offers yet another way of distinguishing between embodiment processes that take place in the background of our experiences (and focused on in Csordas' descriptions) and the kinds of embodiment processes that involves an endeavor of the practitioner and can be specifically addressed as embodied learning.

It should be noted that the distinction between ability and skill presented by Breivik is a conceptual one. Such a distinction does not prevent certain abilities that are practiced under special conditions becoming skills. Motor behaviors like walking and running are, according to Breivik's distinctions, considered basic abilities. These basic abilities are still part of the embodiment process as described in Csordas' work and accordingly attuned, moderated and shaped by how we live our lives with other people, cultures and societies (e.g., Mauss, 1992). Yet the fact that running, for example, is initially considered an ability does not mean that running skills cannot be developed. Depending on the running discipline, these kinds of skills might include, for example, adeptly adjusting one's running speed in relation to other runners in a race, optimizing one's running efficiency in long-distance running, and/or ideally coping with non-injurious pain in middle distance running (e.g., Hockey and Allen-Collinson, 2016; Lev, 2019, 2020; Bluhm and Ravn, 2021).

In the succeeding section I will continue to describe what embodiment processes and incorporation of skill then mean in more concrete terms—and what might be added. I will do so by drawing on my own mountain-biking experience. By way of example, the descriptions presented are thereby intended to highlight further aspects to be kept in mind when discussing how skills are to be considered important aspects of embodied learning.

## Skills And Recreational Mountain-Biking

Recently, I started to learn to mountain bike on different trails in woods and hilly terrain made especially for such activity in Denmark. I have biked since I was a kid. I know how to bike and do so every day to and from the university. Biking is, as such, not a new skill I had to incorporate when I started to mountain-bike. Instead, biking is, using Breivik's conceptual distinctions, more like an ability that I have used as part of everyday life for years and that I have now started to pursue under other conditions. The practice of mountain-biking has demanded a heightened awareness of what this kind of bike “can do,” and it has challenged me to figure out what it takes to bike downhill on relatively steep parts of the trails and on surfaces covered with tree roots. As I bike downhill, I am still in a phase where I concentrate on ensuring that my weight is shifted to the rear of the bike—potentially behind the saddle—and on relaxing the tension in my arms and upper body so I can use the front wheel for steering. Biking uphill over tree roots, I still tend to be keenly focused on one root at a time and often find myself using less effort and losing momentum when I feel the front wheel has just passed one of these relatively big roots. To cope with the different demands and challenges, I have started practicing on “reading” the surfaces and terrain of the trail a bit ahead so that I can adjust the speed and shift to the appropriate gear in preparing to cope with the challenges only a few seconds away. Habitual biking to the University does not demand this same kind of special awareness in terms of the terrain. On the contrary, I simply bike and often think of emails that need answering, etc., in the process. When I am practicing mountain-biking, I rely on my everyday biking skills as well as explore and develop them on new terms. Mountain-biking is an exploratory process of trying things out and listening to good advice while incorporating these specialized biking skills. An experienced mountain-bike instructor will, expectably, be able to set up descriptions and perhaps also taxonomies of the skills that newcomers need to incorporate to become an adept mountain-biker. Such specification would obviously help practitioners reflect on what they have learned and accordingly which kinds of skills they have incorporated. By providing such a field-specific evaluation of the practice, instructors potentially “scaffold” the experience of the practitioner and offer a practice-based framework that can help the practitioner to decide what could be given more attention in future practices (Downey, 2008). At the same time this framework makes visible how mountain-biking skills can be measured by degrees in ways that are recognizable to other mountain-bikers. In other words, the degrees of skills are measured and given value on the contextual condition of the actual field of the physical activity.

In the brief description of learning to mountain-bike, the embodied learning did not derive from the biker focusing on incorporating a specific movement pattern. Rather, in the description of learning to mountain-bike, environmental factors set the scene, so to say. It is by meeting the challenges on the trail—biking downhill, uphill and so on, and coping with the various surfaces—that the mountain-biker develops the kind of skills which specifically characterize mountain-biking. In this sense, incorporating mountain-biking skills is contingent on the practitioners' immersion in the physical, and implicitly social, context of mountain biking. The description of recreational mountain-biking exemplifies processes of embodied learning in which skills are incorporated on the condition of the “relational properties between the learner and the context” (Smith, 2011, p. 265). Accordingly, as also highlighted in Breivik's work, from the outset the skills acquired come with timing and are integrated in complex ways to make up a coordinated whole (Breivik, 2016a, p. 224). This characteristic of skill incorporation is also specifically highlighted by Romdenh-Romluc (2010), in her discussion of Merleau-Ponty's work. Acquiring a specific kind of motor skill does not take place in a contextual vacuum. Instead, “acquiring a motor skill is thus partly a matter of learning to perceive opportunities to exercise it” (Romdenh-Romluc, 2010, p. 77).

At the same time the skillful handling of special kind of actions, such as riding downhill on the trail, involves skilled ways of being aware of and sensing one's movement and the environment. So, in addition to timing and integration, the incorporation of skills also requires practitioners to internalize specialized ways of sensing and being attentive to their bodily sensations, tools in use (e.g., the mountain-bike), other practitioners and the environment (Breivik, 2008, 2014, 2016b). In relation to mountain-biking, these sensations and attention can relate to how riders shift their weight in relation to the bike saddle, how to relax muscle tensions and let the cushioning of the bike work in bumpy sections, reading the trail, surfaces, etc. The growing amount of research focusing on how senses and attention take shape in the process of incorporating skills confirms this and illustrates how the incorporation of skills involves the development of sensing and ways of attending to these sensations. Examples of these descriptions include Gunn Nyberg's work on experiential aspects of embodied learning (Nyberg et al., 2020) and on the somatic grasping of practical knowing of free skiers (Nyberg, 2015); Bluhm and Ravn's analysis of middle-distance runners' expertise in handling their sensation of non-injurious pain while running (Bluhm and Ravn, 2021), and several analyses of how professional dancers use their sensorial awareness when practicing and performing (e.g., Ravn, 2017; Ehrenberg, 2021; Pini and Deans, 2021).

Finally, Breivik (2016a) suggests that, depending on the type of sport and the environment of the sporting activity, talented athletes display a multitude of skills and that these skills might also be mental. Each sports discipline demands a unique combination of mental and motor skills to perform at one's best, come in first or win the game. He emphasizes that we can consider the strategic and/or reflective involvement of the athletes in an activity as mental skills, but only to the degree that these skills “are close to the execution of sports-relative bodily skills” (Breivik, 2016b). Accordingly, considering and setting up tactics and plans for how to play a football match should not be counted as football-playing skills. However, the football player's tactically informed ways of “seeing” and anticipating opportunities in the field while playing and the mountain-bikers' ways of seeing to adjust ahead would count as a mental skill. The latter example is convincingly demonstrated in Christensen et al.'s (2015) analysis of thinking and doing in the case of an expert mountain-bikers' performance practices.

## Skills And Creative Dance

As emphasized in several discussions in learning contexts, expressive and creative dance activities provide learning opportunities that differ from sports and performative activities (Nielsen, 2008; Rustad, 2012; Larsson and Karlefors, 2015; Mattsson and Lundvall, 2015; Mattsson and Larsson, 2021). In a Scandinavian context of physical education, such dance activities are also described by emphasizing practitioner's experience of “movement itself” and as fundamentally different from activities where practitioners are expected to master and perform skills (Engström et al., 2017). As indicated in the introduction to this article, embodiment processes are often highlighted in activities such as expressive and creative dance. However, as should be clear by now, phenomenological descriptions of embodied learning and of the incorporation of skills are not from the outset specifically directed at highlighting certain kinds of pedagogical choices nor specific kinds of physical activity. Fundamentally, the incorporation of skills should be understood as closely related to the ongoing processes of embodiment. Accordingly, it is worth taking a closer look at the kinds of skills that can beat stake in creative dance activities.

For more than two decades, I developed the curriculum and taught creative dance and expressive performance for students studying physical education at the University of Southern Denmark (Ravn, 2001, 2005). In accordance with the field of creative dance activities, involving the movement-analytical framework of Rudolf Laban^3^, some of the courses included explorative phases where experiences and students' descriptions of their experiences played a key role for the learning at stake. At the same time, the course also engaged the students in creating and performing a dance performance. For example, exploring and experimenting with their movement repertoire and the expressive potentials of movement as such constituted part of an embodied learning process in which the students gradually learned new aspects of the skills they had incorporated in sports disciplines, their preferences in movement qualities, and their posture and ways of carrying their body. Adding to this, they also found themselves moving in new ways, such as moving on the floor by rolling through sitting and semi-prone positions, while deliberately using different parts of their body to lead their movement (Ravn, 2005). In this process Laban's work was often crucial in terms of how explorations were guided and became part of the “tools” the students used when exploring and orchestrating selected parts of their movement explorations into a performance. These tools included conceptually based descriptions and differentiation of the dynamic quality of movement and facilitated novel ways for the students to see and sense their own movement.

It would be quite difficult to specify dance-specific sensor-motoric skills which the students incorporated in this course in expressive and creative dance. The students were not expected to learn the genre-based skills of various dance forms related to ballet, flamenco or Irish step dancing, for example. Instead, the embodied learning processes were based on students' explorations of sensory-motor capabilities and possibilities. However, the explorations entailed that they incorporated skilled ways of connecting a Laban approach to the ways they moved and could move. Among other outcomes, they gained a practice-based understanding of how they can choose to shift their way of attending to movement while moving and how to change the dynamic quality of their movement according to intended expressions.

In the fields of sports activities, there are certain built-in logics concerning the way skills are recognized, appreciated and potentially evaluated. Predefined techniques and standardized rules set the framework for these skilled actions—and thereby what it will take to win the game or cross the finish line first. Such logic is highlighted by Breivik when he emphasizes that, from a sporting perspective, “being skillful is thus being able to maximize certainty, minimize physical and mental energy costs, and minimizing time used” (Breivik, 2016a, p. 224). Achieving a successful performance “presupposes physical skills that are integrated and coordinated relative to a time horizon, a context and in view of a task or goal” (Breivik, 2016a, p. 225). The ways that athletes perceive a sporting environment as “inviting” them to interact in these activities are guided by the logic of competition.

Turning to expressive and creative dance activities, one meets other kinds of built-in logic of how the relevant kinds of skills are recognized and appreciated. The kind of skills incorporated are to be practiced and comes in degrees, in accordance with Breivik's definition for skills. On the contextual terms of expressive and creative dance, students can become even better at fine-tuning and shifting the dynamic quality of a movement sequence. Central to the kinds of skills incorporated in dance is, for example, the way the practitioner senses, is aware, and specifies the quality of the actual movement while moving. The incorporation of these skills does not first connect to a specific technique or to a specific kind of sensory-motoric movement pattern. Rather, the skills acquired in creative dance are best described according to the novel ways and variations of how different kinds of movement patterns are carried out. Thus, if we use a sequence of moving/rolling through sitting and laying positions on the floor, a skilled way of doing so could, for example, include deliberate shifts in the way the weight factor (or force)^4^ is invested while moving.

Skills are recognized on field-dependent conditions. Thus, for obvious reasons, the kind of complexity and coordinated whole that characterizes the skills developed in creative dance are different from the complexity and coordinated whole characterizing the kind of skills identified in relation to many sports activities. Skills—and the degree of these skills relevant to the fields of creative dance practices—are measured on contextualized scales of expressivity and often in a way, so that this scale is directly relevant to practitioners' lived experiences (e.g., Ravn and Elmose-Østerlund, 2019).

## Attunement to Interaction

Embodied learning in expressive and creative dance practices can be described according to skills. At the same time, however, this field of physical activity prompts considerations of other aspect of embodied learning. As implicitly indicated, engaging in movement explorations and experimentations is often based on improvisation. In this section, I consider what these improvisational practices might add to descriptions of embodied learning in physical activity. I begin by specifying what improvisation “is” as well as emphasize that, like skills, improvisation unfolds as field-dependent practices.

As I have argued in depth elsewhere (Ravn, 2020), any dance can be considered improvised, albeit in different ways and degrees. Within the contexts of the different fields of dance practices, the dance might be characterized as choreographed or improvised. However, no matter how it is addressed and appreciated—or not—any dance will have an openness and spontaneity built into the process of enacting it (Ravn, 2020). I do not thereby mean to suggest that we erase or ignore the special kind of expertise characterizing dance improvisers—on the contrary. My point is rather that the dancers who specifically address improvisation are expected to specifically deal with, and potentially highlight how the openness of the situation can be realized in ways that appreciate the opportunity to move spontaneously.

Casting a glance on the different fields of improvisational dance practices, one immediately realizes that the different kinds of practices present multiple ways of addressing the openness and spontaneity of the dancing. In some improvisational practices, such as contact improvisation (Engelsrud, 2007), Argentinean tango (Ravn, 2019) and Mambo (Goldman, 2010), specific kinds of movement patterns and techniques form the framework for how practitioners are expected to move. In these kinds of dance improvisation, practitioners are explicitly expected to also have trained and incorporated certain skills to be able to participate in the activity. In Argentinean tango and Mambo, dancers are expected to improvise based on a certain kind of movement pattern (specific steps and a combination of these) whereas contact improvisers are expected to have gained differentiated experiences of lifting and falling^5^. In artistic fields of contemporary dance, improvisation is presented and discussed as an expert capacity (e.g., Albright and Gere, 2003; De Spain, 2014) that involves and links to certain performance traditions and which demands both training and preparation (Ravn et al., 2021). Thus, my point here is, that different kinds of experiential background and skills underlie the kind of improvisation strived for in a given field of dance practice (Midgelow, 2019; Ravn, 2020). Enacting incorporated skills is not in opposition to the practicing of improvisation. At the same time, however, we would miss the point if we ended up focusing on descriptions of dancers' capability to improvise by evaluating the kind of skills incorporated. Something else and more is also at stake when improvising.

Based on the analysis of two professional artists (dance and music) I have recently pointed out in collaboration with philosopher Simon Høffding (Ravn et al., 2021), that improvisers seem both to engage in a spontaneous “letting things happen” and exercising specialized forms of mental and bodily skills. Experienced from the perspective of the improvisers, the actions performed while improvising “are vested in the interactive dynamic itself” (Ravn et al., 2021). In different ways, an improviser oscillates in sophisticated ways between assuming and relinquishing control of the dancing taking place. Accordingly, we (Høffding and Ravn) have suggested that the improvisation taking place is based on the improvisers interacting or setting up an encounter with different kinds of interactive possibilities. These possibilities (or forces) might address, for example, the movement and actions of another dancer but not only. They might also address the music and/or the musician(s), the audience, imaginative landscapes and sensed energies (e.g., Ravn, 2017, 2020; Høffding and Snekkestad, 2021; Ravn et al., 2021). Notably, even in improvisations where the dancer is the sole performer on stage, such interactive forces seem to be at stake and play a fundamental role for the improvisational practice taking place (Ravn, 2017, 2020; Høffding and Snekkestad, 2021; Ravn et al., 2021). Reading through the dance scholarly work on improvisation in contemporary dance practices, this account of improvisation resonates with several descriptions presented from within the fields of dance research. For example, in her relatively general introduction to improvisation related to contemporary dance practices, leading dance scholar Susan L. Foster^6^ emphasizes that improvisation with other dancers is not a matter of either leading or following, but of “moving with, and being moved by another body. One's body weight and momentum flow into and with another body's shaping and trajectory making a double bodied co-motion” (Foster, 2003, pp. 7–8).

Contemporary dancers who are professionally engaged in performing choreographed dance pieces as well as in performing in improvisation-based dance events emphasize that being vested in the interactive dynamic of improvisation demands another kind of attunement. They *attune* their way of being attentive to others differently when engaging in the improvisation of a performance compared to dancing a choreographed dance piece (Ravn, 2009, chapter 6). There is a kind of shift or shifting in how they direct their attention to the actions of their own moving body vs. the interaction taking place in the here and now. As one dancer describes “in a set choreography he experiences that he is “measuring space,” while in improvisation he “is part of space”” (Ravn, 2017, p. 66).

A similar kind of distinction between being focused on the action of one's own body vs. being with the interactions of the here and now is described by four tango leaders who were interviewed about their way of interacting with the follower and improvising the tango (Ravn, 2017). Each of the tango dancers in their own way highlighted that there is a difference between exercising a certain pattern of steps alone or with their partner and improvising a tango based on these patterns. When the improvising of movement and steps take shape in and through the dynamic created between the two tango dancers, they experience that they dance with their partner *through* the interaction. For example, one of the interviewed tango dancers describes that when improvising, “I am also in her body,” adding that when the good dance happens, and the improvisation really works, “I have the experience that it “it dances me”” (Ravn, 2017).

Relating to and forming part of interactive forces as these unfold in the here and now is not a straightforward task. It demands practice to attune oneself differently to the interaction and to actively realize the possible openness and spontaneity the dancing. Thus, it is worth asking if it is relevant or even possible to consider improvisers' ways of attuning themselves to interactions as simply another kind of mental skill. In other words, can we think of the kind of attunement which different dancers describe, as a special kind of skills that simply contributes to and nuances Breivik's description of skills and to how skills are integrated in complex ways? Trying to do so would, however, present serious problems in relation to how skills are defined by Breivik as well as Leder (1990). It is worth reiterating that skills, as emphasized in Breivik's definition, come with timing and integration and are part of a context. Performing a skill involves, *per se*, that the practitioners adjust the way the skills are performed in response to environmental factors, other practitioners, and so on. However, listening to how improvisation is vested in the interactive dynamic itself and how dancers might experience moments in improvisation as if being moved through the interaction (i.e., other dancers, “space,” audience, and so on) hardly reflect the way “adjustments” and “responses” are used to describe the way skills are part of a complex whole. Descriptions and analyses of skills are focused on how specific kinds of movement are enacted by a practitioner. Using such focus, we can discuss if and how the practitioner acts in competent ways according to the contextual demands of the physical activity in focus. In descriptions of embodied learning, the notion of skill thereby invites focusing on the actions of the agent. By comparison, descriptions of improvisational practices prompt a shift in focal point to focusing on interactions, rather than on the actions of agents. Accordingly, the aspect of embodied learning in focus will also shift toward interactions and the way practitioners engage in, and attune to, interactions as these unfold in the here and now of the activity.

I suggest that we recognize improvisers' field-dependent ways of being with interactions as based on their capability to attune to being moved through interactive forces and that we consider this capability to attune to interaction as yet another aspect of embodied learning, an aspect which develops in complementary ways to the incorporation of skills. In the following section I connect this suggestion to recent phenomenological considerations on how agent, interactions and situation are co-constituted and thereby add further considerations to the description of what it means to attune to interactions of actual practices.

## Attunement to Interactions of Situations

Drawing on Dewey's notion of the situation, Gallagher (2020) highlights that for any action taking place we should distinguish between context (or circumstances) and situation. Unlike context, the situation includes the agent in a way in which situation and agent are co-defined (Gallagher, 2020, p. 13). The agent participating and acting in a practice cannot step out of the situation without affecting it. In the words of Gallagher, “I can point to a relevant part of the environment, but I cannot strictly point to the situation because my pointing is part of the situation” (Gallagher, 2020, p. 13). Agent and situation are not only co-defined but dynamically constituted throughout the actions taking place. Gallagher presents a phenomenologically based account of how the intentional formation of the agent forms part of a complex interplay between the agents' intentional engagement and the situation they form part of. His account of action and interaction thereby presents a phenomenological basis for suggesting that if we are to understand practitioners' ability to act in relevant ways in ongoing practices, we should also pay attention to the situated conditions. Improvisational practices highlight how practitioners can attune themselves to the interactions constituting the situation.

Gallagher (2020, p. 10) describes affordance as the specification of a relation between an agent and some aspect of the environment and, in accordance, social affordances as the relation between an agent and some aspects of the social. He highlights that a situation has a structure that in many ways is comparable with affordances in the sense that, like “affordance and actions,” “situations and actions” are co-constituted (Gallagher, 2020, p. 13). In that sense, a situation contains a variety of affordances which the agent, or practitioner, will have to relate to and form part of with different preferences. Following Gallagher's account, improvisers attuning to interactions, as these take shape in the here and now, should not be misunderstood as “just giving in” and letting situational dynamics do their job. Being moved through the movement of the interactive forces—such as other dancers, imaginary forces, music, audiences, etc.—involves differentiations in the way the practitioners attune to being aware of and being with specific kinds of affordances offered in the situation.

Practitioners' skilled actions and interactive attunement have different kinds of importance to how well they participate and/or perform in a given physical activity. Activities like mountain-biking require practitioners to incorporate specific skills so that they find ways to be in control of their biking actions on the trail. The actions of the agent are thus at the foreground of the way embodied learning is described. By comparison, the descriptions and examples of competent and expert improvisers by context specifically address the interaction and the practitioners' ability to attune to these and form part of the interaction. In these practices, interaction is at the forefront while skills and skilled action is pushed into the background when describing the embodied learning at stake.

## Conclusion

This article has been motivated by recent pedagogical discussions in the field of physical education. In these discussions, embodiment processes tend to be primarily connected to non-sporting physical activities that favor critical reflection on self-identity and/or exploration of novel movement activities while partly ignoring the embodiment processes connected to the acquisition of skills (e.g., Larsson and Karlefors, 2015; Standal and Aggerholm, 2016; Aggerholm et al., 2018; Aartun et al., 2020). Skills and skilled performance tend to be presented primarily in relation to sporting activities and thereby partly in opposition to activities that invite reflection and exploration (Engström et al., 2017). The main argument in this article is that if we base analyses of practicing a physical activity on phenomenological descriptions of embodiment, the incorporation of skills present important aspects to be considered in any kind of physical activity, including, for example, explorative and reflective approaches characterizing expressive and creative dance activities. I would like to emphasize that I have not thereby argued against the pedagogical discussions emphasizing that expressive and creative dance activities invite novel experiences and that these might facilitate students' reflections on the body, movement and sense of self. However, I have argued that the kind of skills at stake in activities such as expressive and creative dance should not be ignored just because the skills are different compared to the sports-related skills.

Drawing on phenomenological descriptions of embodiment, I have indicated that the process of incorporating skills is grounded in the ongoing transformation of embodiment processes and includes concurrent developments of perceptual fields. In concrete terms and as further exemplified through the descriptions of mountain-biking, skills form part of a complexity and are from the outset context sensitive. Actual skills of relevance to the kind of physical activity in focus are to be recognized on field dependent conditions. They involve sensory dimensions, and certain ways of being aware of one's body and the environment. Handling skills as closely specified according to certain movement techniques could definitely be the case if we look into sporting activities, and it is indeed the case when following Breivik's descriptions of the role of skills in sports achievements. However, in the expressive and creative dance activities described, the skills at stake can be described according to the ways in which practitioners are capable of differentiating and shifting between qualitative dynamics when moving and do not explicitly mean that certain movement techniques are learned. However, throughout the article, I have moved between phenomenological accounts of embodiment processes and descriptions of actual practices. Phenomenological accounts and related theoretical discussions have paved the way for the argument. At the same time, however, I have used my research background, which is based in the analyses of actual physical activities (especially different kinds of dance practices), to exemplify accounts and constructively contribute to enhance the description of embodied learning. In that sense, the argument has been driven forward through a dialectical movement between phenomenological accounts (of embodiment, skills, and action and interaction) and descriptions of actual (non-sporting) practices.

In the latter part of the article, I suggest that, in addition to skills, we should consider practitioners' capability to attune to interaction as an aspect of embodied learning, an aspect which works in complementary ways to the incorporation of skills. This theoretical proposition is based on several works about improvisational practices spanning quite different fields of dance activities. Gallagher's (2020) account of action and interaction presents a phenomenological ground for supporting the description of attunement to interaction presented. The suggestion is based on dance practices, but it is inherent in the suggestion and supported by the dialectical nature of the underlying argumentation that such attunement will also be of relevance when analyzing the embodied learning at stake when practicing other kinds of physical activity. I suggest that, along with incorporating skills, practitioners potentially develop the capability to attune themselves to be moved by the interaction as for example the movement of the other practitioners practicing the activity. Accordingly, when investigating, discussing or analyzing embodied learning related to playing football, riding a mountain-bike, or improvising in dance, for example, one should consider the specific kinds of skills incorporated as well as the kind of attunement to interaction involved in the given physical activity.

## Data Availability Statement

The original contributions presented in the study are included in the article/supplementary material, further inquiries can be directed to the corresponding author.

## Author Contributions

The author confirms being the sole contributor of this work and has approved it for publication.

## Conflict of Interest

The author declares that the research was conducted in the absence of any commercial or financial relationships that could be construed as a potential conflict of interest.

## Publisher's Note

All claims expressed in this article are solely those of the authors and do not necessarily represent those of their affiliated organizations, or those of the publisher, the editors and the reviewers. Any product that may be evaluated in this article, or claim that may be made by its manufacturer, is not guaranteed or endorsed by the publisher.

## Footnotes

^1^Merleau-Ponty himself uses the notion “instrument” in relation to the body on occasion. The notion of “instrument” refers to the materiality of the body preconditioned in certain ways and lived. The notion is not to be understood as referring to a new kind of objectification or instrumentality.

^2^In Breivik's description, abilities work in taken for granted ways. For clarification, I find it worth adding that changes in our state or mode of being (e.g., being very tired, drunk or the like) might influence the way these abilities work for us. However, such kinds of changes in how abilities support our behavior should not be mistaken as indications of certain degrees of how these abilities are “mastered”.

^3^In the Laban-Bartinieff version, the framework is built around the movement concepts body, effort, space, and shape. (See for example https://labaninstitute.org/ For an overview and discussion of Laban's work, see McCaw, 2011).

^4^As one of the four effort factors, weight can be described in the tension between light and strong investment of weight. This dynamic quality of movement is often deemed similar to using light or strong force, respectively.

^5^The kind of falling expected has been inspired by the kind of falling trained in aikido (Novak, 1990).

^6^Foster is one of the most influential dance historians of today. Based in the genealogical and discursively oriented analysis of different kinds of dance practices, her work spans analyses of different kinds of historical, contemporary and new dance forms and theoretical conceptual discussions. For a complete list of her work, see https://www.wac.ucla.edu/people/faculty/susan-leigh-foster.
